# Patterns of recurrence in anal cancer: a detailed analysis

**DOI:** 10.1186/s13014-020-01567-7

**Published:** 2020-05-27

**Authors:** Martin P. Nilsson, Erik D. Nilsson, Anders Johnsson, Otilia Leon, Adalsteinn Gunnlaugsson, Jonas Scherman

**Affiliations:** 1 Division of Oncology and Pathology, Department of Clinical Sciences, Lund University, Lund, Sweden; 2 Department of Hematology, Oncology and Radiation Physics, Skåne University Hospital, Lund, Sweden; 3 Department of Clinical Sciences Malmö, Lund University, Lund, Sweden; 4 Radiation Physics, Department of Hematology, Oncology and Radiation Physics, Skåne University Hospital, Lund, Sweden

**Keywords:** Anal cancer, Anal carcinoma, Radiotherapy, Recurrence, Para-aortic, Ano-inguinal lymphatic drainage

## Abstract

**Background:**

Anal cancer is a rare disease, which might be the reason for the “one size fits all” approach still used for radiotherapy target contouring. To refine and individualize future guidelines, detailed and contemporary pattern of recurrence studies are needed.

**Methods:**

Consecutive anal cancer patients, all treated with curative intent intensity-modulated radiotherapy (IMRT), were retrospectively studied (*n* = 170). Data was extracted from medical records and radiological images. Radiotherapy planning CT’s and treatment plans were reviewed, and recurrences were mapped and categorized according to radiation dose.

**Results:**

The mean dose to the primary tumor was 59.0 Gy. With a median follow-up of 50 months (range 14–117 months), 5-year anal cancer specific survival was 86.1%. Only 1 of 20 local recurrences was located outside the high dose (CTVT) volume. More patients experienced a distant recurrence (*n* = 34; 20.0%) than a locoregional recurrence (*n* = 24; 14.1%). Seven patients (4.2%) had a common iliac and/or para-aortic (CI/PA) recurrence. External iliac lymph node involvement (*P* = 0.04), and metastases in ≥3 inguinal or pelvic lymph node regions (*P* = 0.02) were associated with a 15–18% risk of CI/PA recurrence. Following chemoradiotherapy, 6 patients with recurrent or primary metastatic CI/PA lymph nodes were free of recurrence at last follow-up. The overall rate of ano-inguinal lymphatic drainage (AILD) recurrence was 2 of 170 (1.2%), and among patients with inguinal metastases at initial diagnosis it was 2 of 65 (3.1%).

**Conclusions:**

We conclude that other measures than increased margins around the primary tumor are needed to improve local control. Furthermore, metastatic CI/PA lymph nodes, either at initial diagnosis or in the recurrent setting, should be considered potentially curable. Patients with certain patterns of metastatic pelvic lymph nodes might be at an increased risk of harboring tumor cells also in the CI/PA lymph nodes.

## Background

Squamous cell carcinoma of the anal region (anal cancer) is a rare malignancy that is usually treated with chemoradiotherapy (CRT). This is an effective treatment curing a majority of the patients [1–3]. Importantly, after successful CRT many patients suffer from late side-effects with a negative impact on the quality of life [4–6]. Therefore, for each individual patient, a careful balance must be struck between the chance of cure and the risk of severe toxicity.

Current international guidelines do not recommend different cranial borders of the elective clinical target volume (CTV) based on the risk of recurrence [7–9]. This is in contrast to guidelines for squamous cell carcinomas of other primary locations. In oropharyngeal carcinoma, the extent of the elective CTV varies with TNM stage and primary tumor location [10], and in cervical carcinoma, the cranial border varies with the risk of para-aortic recurrence [11]. Anal cancer is a rare disease, which is most likely the reason for the “one size fits all” approach used until now. According to RTOG, as well as Australian guidelines, the cranial border of the elective CTV should be where the common iliac vessels bifurcate into external/internal iliacs (approximated boney landmark: sacral promontory). The cranial border according to UK guidelines is 20 mm above the inferior aspect of the sacroiliac joint or 15 mm above the most superior aspect of the gross tumor, whichever is most superior.

To refine treatment recommendations, detailed and contemporary patterns of recurrence studies are needed. The long term goal is that future anal cancer contouring guidelines should differentiate between patients at different levels of risk – for some patients, the elective CTV might be reduced, and for others, it might be expanded. Current UK guidelines, where the mesorectal volume is reduced in the absence of mesorectal disease, and omission of elective nodal stations if considered for some good prognosis T1N0 tumors, is a first step in that direction.

From a population-based institutional database of consecutive anal cancer patients, we selected patients treated with curative intent intensity-modulated radiotherapy (IMRT). A study of patterns of recurrence was undertaken, focusing mainly on: (1) the frequency and location of locoregional and distant recurrences; (2) the risk of – and factors associated with – common iliac and/or para-aortic (CI/PA) recurrence; (3) the risk of recurrence in other regions of interest, such as the ano-inguinal lymphatic drainage (AILD) [12–16], the sacral hollows, and the part of the inguinal area that is located posterolateral to the deep vessels [17].

## Material and methods

### Study population

**Fig. 1:**
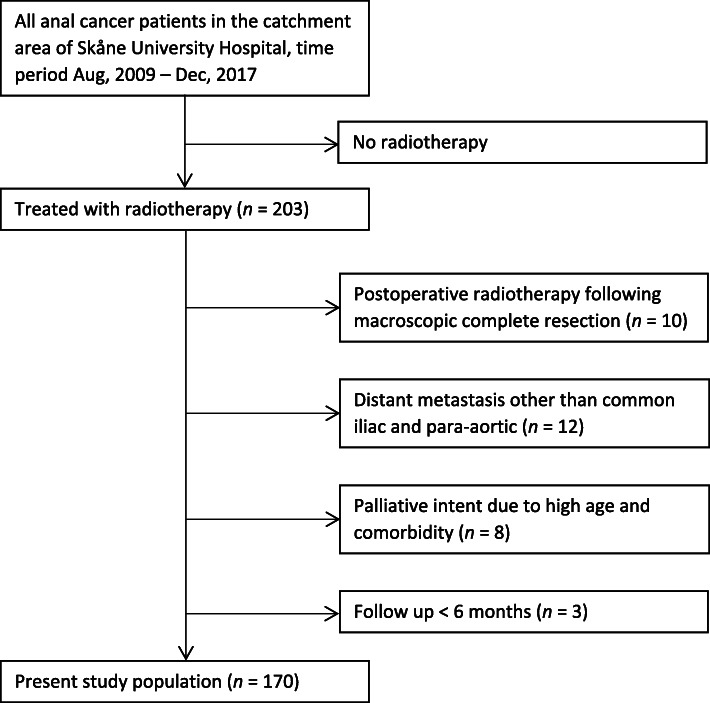
Flowchart of the study population

All patients with anal cancer treated with radiotherapy at the Skåne University Hospital, Lund, Sweden, during the time period Aug, 2009 – Dec, 2017 were selected from an institutional database (*n* = 203). From Aug, 2009 and onwards, IMRT was used for all anal cancer patients in Lund. Patients with palliative intent treatment or distant metastasis at diagnosis were excluded; however, patients with isolated metastatic CI/PA lymph nodes were not excluded. Following exclusion of patients without macroscopic tumor left after primary surgery, and patients with < 6 months of follow up, 170 patients remained and constituted the present study population (Fig. 1). Skåne University Hospital serves a catchment area of 1.9 million inhabitants, and there are no other radiotherapy departments treating anal cancer in the region. Therefore, the study population is a consecutive population-based series.

### Treatment and follow-up

Treatment was according to Swedish national guidelines, which are relatively detailed regarding chemotherapy and radiation dose, but not detailed regarding delineation [18]. Before 2017, patients with T1–2(< 4 cm)N0 (Group A) were treated with IMRT 42.0 Gy in 21 fractions to the tumor and the elective lymph node stations, followed by a boost of 12.0 Gy in 6 fractions to the tumor. Patients with T2(≥4 cm) or T3–4 or N+ (Group B) were treated with IMRT 46 Gy in 23 fractions, followed by a boost of 14.0 Gy in 7 fractions to the primary tumor and pathologic lymph nodes. From 2017, a simultaneous integrated boost (SIB) technique was used. For Group A, the dose was 41.6 Gy in 27 fractions to the elective CTV and 54.0 Gy to the tumor. For Group B, the dose was 41.6 Gy in 27 fractions to the elective CTV, 50.5 Gy to pathological lymph nodes < 4 cm, and 57.5 Gy to the tumor and pathological lymph nodes ≥4 cm. All patients were treated in the supine position. Radiotherapy was given without planned treatment breaks. It was recommended - but not compulsory - that the contouring of the elective CTV should in line with the RTOG guidelines, with some minor local variations. For instance, the entire ischiorectal fossa was included for most patients, and the sacral hollows were usually not included (Table 1). Regarding the cranial border, the common iliac bifurcation was used. However, since the sacral promontory is often considered a bony landmark corresponding to the common iliac bifurcation, some clinicians continued to use that landmark, even in the IMRT era. The margin used from CTV to PTV (planning target volume) at Skåne University Hospital during the period of interest was 7–8 mm.

**Table 1:** Patient, tumor, and treatment characteristics

	*n* (%)
**Age at diagnosis (years)**	
Mean; SD	64.4; 9.3
**Female gender**	137 (80.6)
**Staging**	
CT	170 (100)
PET	165 (97.1)
MRI	156 (91.8)
**Tumor localization**	
Anal canal	32 (18.8)
Anal canal + rectum^a^	48 (28.2)
Anal canal + perianal^b^	52 (30.6)
Anal canal + rectum + perianal	30 (17.6)
Perianal	5 (2.9)
Rectum	3 (1.8)
**T stage**^c^	
1.	14 (8.2)
2.	83 (48.8)
3.	35 (20.6)
4.	38 (22.4)
**Lymph node metastasis, N +** ^c^	88 (51.8)
**Site of lymph node metastasis**	
Inguinal	65 (38.2)
Internal iliac	20 (11.8)
External iliac	21 (12.4)
Mesorectal^d^ or presacral	33 (19.4)
Common iliac or para-aortic	4 (2.4)
**Radiation dose, primary tumor**	
Mean; SD (Gy)	59.0; 2.8
52–55 Gy	28
56–60 Gy	125
61–64 Gy	17
**Radiation dose, lymph node metastasis**	
Mean; SD (Gy)	57.4; 4.8
40–50 Gy	16
51–55 Gy	10
56–60 Gy	58
61–64 Gy	4
**Radiation dose, elective CTV**	
Mean; SD (Gy)	44.7; 4.9
40–42 Gy	38
46 Gy	120
48.6 Gy	12
**Margin from GTVT to CTVT**	
< 1,5 cm	9 (4.7)
1,5 cm	132 (77.6)
> 1,5 cm	29 (17.1)
**Margin from GTVN to CTVN**^e^	
≤ 0,5 cm	38 (45.2)
0,6–0,9 cm	25 (29.8)
≥ 1,0 cm	21 (25.0)
**Included in elective CTV**^f^	
Inguinal	167 (98.2)
Internal iliac	168 (98.8)
External iliac	157 (92.4)
Presacral	168 (98.8)
Mesorectal	169 (99.4)
Ischiorectal fossa	154 (90.6)
Sacral hollows	13 (7.6)
**Superior border of elective CTV**	
> 2 cm above iliac bifurcation^g^	8 (4.7)^h^
Within +/− 2 cm from iliac bifurcation	114 (67.1)
2,1–4 cm below iliac bifurcation	33 (19.4)
> 4 cm below iliac bifurcation	15 (8.8)
**Radiation treatment time**	
Mean; SD	41.5; 4.4
Median; range	42.0; 35–69
**Radiation technique**	
IMRT	14 (8.2)
Tomotherapy	36 (21.2)
VMAT	120 (70.6)
**Induction chemotherapy**	10 (5.9)^i^
**Concomitant chemotherapy**	152 (89.4)
2 cycles	103^j^
1 cycle	49^k^
+ cetuximab^l^	8
Dose reduction of chemotherapy	31
**Stoma before start of radiotherapy**	23 (13.5%)^m^
**Salvage surgery**	
Indication locoregional recurrence	16 (9.4%)
Indication severe toxicity	5 (2.9%)

*Abbreviations*: *SD* Standard deviation, *CT* Computed tomography, *PET* Positron emission tomography, *MRI* Magnetic resonance imaging, *CTV* Clinical target volume, *GTV* Gross target volume, *IMRT* Intensity modulated radiation therapy, *VMAT* Volumetric modulated arc therapy, *FUMI* Fluorouracil + mitomycin C

^a^rectum = tumor extension above puborectalis muscle

^b^perianal = tumor extension outside the anal verge

^c^TNM8

^d^Superior rectal included in mesorectal

^e^Lymph node metastases were not boosted in 4 patients

^f^> 80% of region covered in elective CTV to count as “included”

^g^Common iliac artery into external and internal iliac arteries

^h^Including 4 patients with common iliac or para-aortic metastasis

^i^9 of 10 also received concomitant chemotherapy

^j^Fluorouracil + mitomycin C (*n* = 102) or platinum based

^k^Fluorouracil + mitomycin C (*n* = 41) or platinum based

^l^Prospective Phase I study (Leon et al, Eur J Cancer. 2015;51:2740–6)

^m^4 of 23 eventually reversed

For Group A, concomitant chemotherapy consisted of one cycle of mitomycin C (10 mg/m^2^ day 1) and 5-fluorouracil (1000 mg/m^2^ days 1–4) [19]. For Group B, concomitant chemotherapy consisted of two cycles of the same regimen, given days 1–4 and 29–33.

Clinical evaluation was performed every 3 months for 2 years, and every 6 months to 5 years. For most patients (156 of 170, 91.7%), a PET-CT was performed approximately 3–4 months after the end of treatment. After that, radiological evaluation was only performed as indicated by clinical symptoms, which might have delayed the detection of some recurrences.

### Data collection

Data on patient, tumor, and treatment characteristics, as well as recurrence and other follow-up data, were extracted from the medical records. Information on T and N stage was collected from radiology reports, clinical examination, and multi-disciplinary team meetings; in case of discrepancies, the latter took precedence. To determine the exact site of metastatic lymph nodes, the diagnostic MRI and/or PET-CT were retrospectively reviewed. Every patient’s radiotherapy planning CT was retrospectively evaluated by a radiation oncologist (MPN), and the following variables were recorded: margin from GTVT (GTV; gross tumor volume) to CTVT; margin from GTVN to CTVN; and extent of the elective CTV.

Every patient with a local recurrence (LR) or a regional recurrence (RR) had a diagnostic PET-CT performed at the time of recurrence. This PET-CT was manually registered with the planning CT, based on soft tissue anatomy, in order to be able to determine whether the recurrence was within the high dose (CTVT) volume or not. Additional findings from clinical examination and pathology analysis of salvage surgery specimens also contributed to the assessment.

**Fig. 2:**
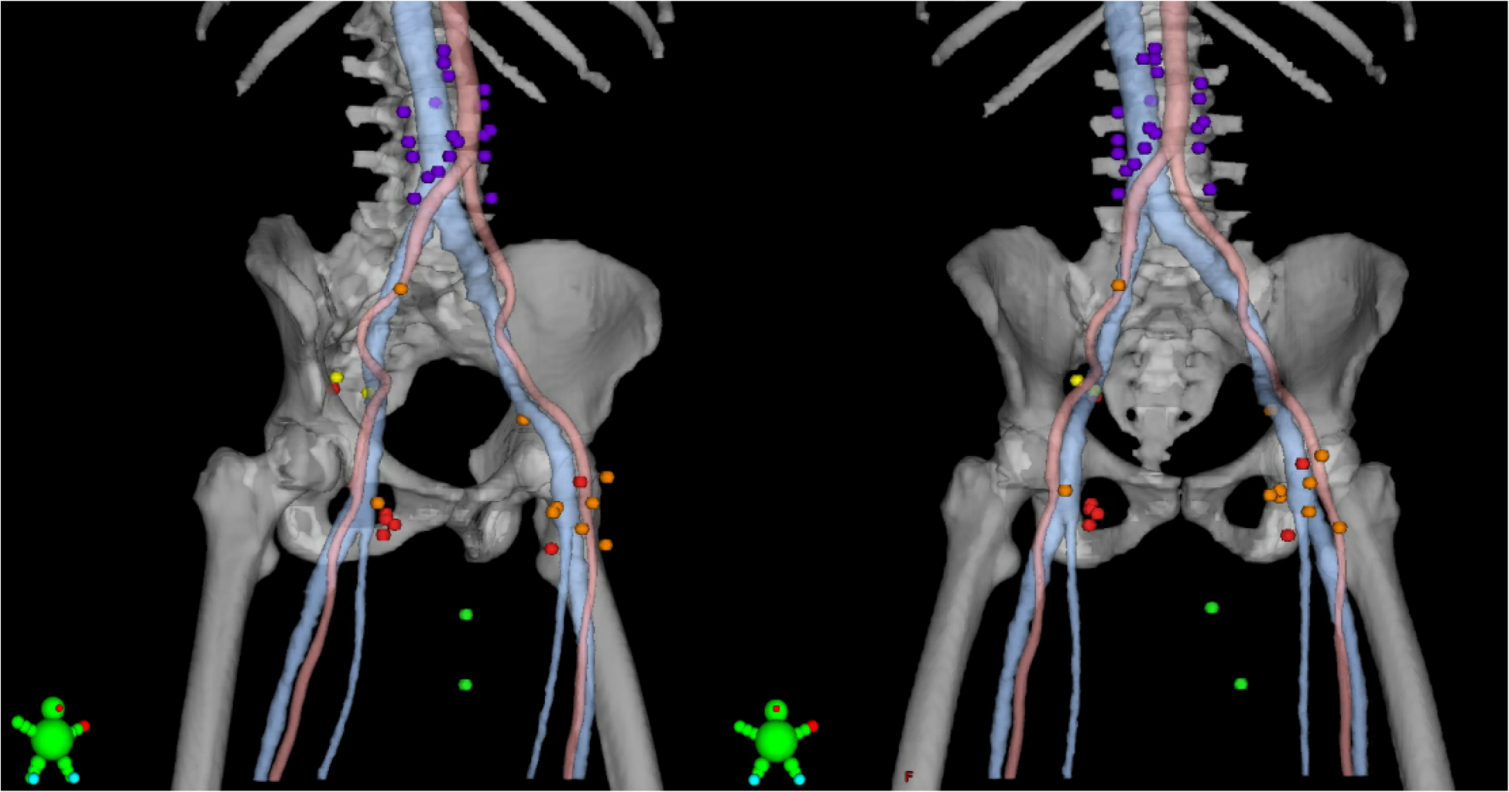
Regional, ano-inguinal lymphatic drainage (AILD), and common iliac/para-aortic (CI/PA) recurrences in 17 patients. Recurrence in GTVN (red; 7 patients, 7 lymph nodes); within elective CTV (orange; 7 patients, 10 lymph nodes); outside elective CTV but within pelvis or inguinal (yellow; 1 patient, 2 lymph nodes); AILD (green; 2 patients, 2 metastases); CI/PA (purple; 7 patients, 20 lymph nodes). Blue, inferior vena cava, external iliac, femoral, and great saphenous vein. Center of lymph node mapped with a 9 mm sphere in a standard anatomy reference CT

RR and recurrences in the AILD, as well as CI/PA recurrences, were mapped on a standard anatomy reference CT (Fig. 2). The center of each lymph node was mapped mainly based on its relation to major arteries and veins. Specifically, in the cranio-caudal direction, inguinal recurrences were mapped based on the level of the saphenous junction, defined as the first CT-slice with visible fat separating the saphenous vein and the femoral vein.

### Endpoints and statistical analysis

Since the number of CI/PA events was so low, logistic regression analyses were not deemed meaningful to carry out. Instead, factors of potential importance for CI/PA recurrence were compared between patients without, and patients with CI/PA recurrence, using crosstabs. Statistical significance was assessed with chi-squared test or Fisher’s exact test, as appropriate.

Complete response (CR) was defined as the absence of disease at the site of the primary tumor and regional lymph nodes within 6 months from the end of radiotherapy. Persistent disease was failure to achieve a CR. LR was defined as persistent disease or recurrence at the site of the primary tumor. RR was defined as persistent disease or recurrence elsewhere in the pelvis or inguinal nodes. Locoregional recurrence (LRR) was defined as either a LR or a RR. Distant recurrence (DR) was defined as recurrent disease outside the pelvis or inguinal nodes, independent of locoregional status. Accordingly, a DR occurring after a LRR was also included. Following a DR, patients could continue to contribute data on LR and RR, provided that the information regarding locoregional status was still deemed reliable; otherwise, they were censored for locoregional endpoints at the last date of reliable data on locoregional status.

Three-year and five-year outcomes were estimated by the Kaplan-Meier method. Follow-up and time to event was defined from the date of diagnosis, except for LR and RR, where it was defined from the end of radiotherapy. For analyses or recurrence, patients were censored at last follow-up or death. Events for disease free survival (DFS) were any recurrence and death from any cause, and patients were censored at last follow-up. Events for anal cancer specific survival (ACSS) were death from anal cancer (including 2 patients with late grade 5 toxicity), and patients were censored at death from other causes or last follow-up. Events for overall survival (OS) were death from any cause, and patients were censored at last follow-up. Analyses were based on follow-up information through Sept 9, 2019.

All significance tests were 2-sided, and *P* values < 0.05 were considered significant. Statistical analysis was conducted using SPSS version 24 (SPSS Inc., Chicago, Illinois, USA).

## Results

### Treatment and survival

One-hundred and seventy consecutive patients with non-metastatic anal cancer were treated with curative intent IMRT (concurrent chemotherapy; 89.4%). A majority were staged with PET-CT (97.1%) and MRI (91.8%), and 51.8% were lymph node positive. Most patients received a 40–48 Gy elective dose to internal iliac (98.8%), external iliac (92.4%), presacral (98.8%), mesorectal (99.4%), and inguinal (98.2%) lymph node stations (Table 1).

The mean dose to the primary tumor was 59.0 Gy, and 157 of 170 patients (92.4%) achieved a CR. With a median follow-up of surviving patients of 50 months (range 14–117 months), the 3-year survival outcomes were: OS (88.9%), ACSS (91.1%), and DFS (74.1%). The 5-year survival outcomes were: OS (79.9%), ACSS (86.1%), and DFS (65.9%). DFS according to TN stage (same subgroups as in Martin et al. [20]) is presented in Table S1. At last follow-up, 23 patients had died from anal cancer, and 13 had died from other causes.

### Local recurrence

At a median of 17 months (range 12–37 months) from the end of radiotherapy, 7 patients were diagnosed with a LR. Together with 13 cases of persistent disease, in total 20 of 170 patients (11.8%) had a LR. It was deemed that 19 of 20 LR were located in, or originated from, the high dose (CTVT) volume, i.e. in-field recurrence. The mean dose to the primary tumor in these 19 in-field recurrences was 58.8 Gy (range 54–64 Gy). Only one LR was located outside the CTVT volume: at its cranial border in the lower rectum. The rate of LR was not different for various margins used from GTVT to CTVT; < 1.5 cm (11.1% LR), 1.5 cm (12.1% LR), and > 1.5 cm (10.3% LR, *P* = 0.98).

### Regional recurrence

At a median of 8 months (range 2–37 months) from the end of radiotherapy, 12 of 170 patients (7.1%) were diagnosed with a RR in 16 separate sites. The most common site was inguinal (*n* = 12), followed by external iliac (*n* = 3), and internal iliac (*n* = 1). No presacral or mesorectal RR were seen. The prognosis for patients with RR was very poor; at last follow-up, eight had died from anal cancer, two were alive with metastatic anal cancer, and one was waiting for further salvage surgery. Only one was alive with no evidence of disease.

In 7 cases, the RR was located in a GTVN; i.e., a lymph node that was deemed pathologic at the time of diagnosis (Fig. 2). One patient with a superficial inguinal metastasis was treated with Tomotherapy without the use of a bolus; in that case, uncertainties regarding superficial dose coverage might have contributed to the recurrence.

Seven patients had a RR within the elective CTV. Six of 7 also had either a LR or a GTVN RR, indicating that these tumors were radioresistant. The last patient first experienced a common iliac recurrence 2 years after the end of radiotherapy, and then had a RR just below the cranial border of the elective CTV another year later. Taken together, no patient had an isolated RR within the elective CTV.

### Distant recurrence

At a median of 10 months (range 5–81 months) from the date of diagnosis, a DR occurred in 34 of 170 patients (20.0%). The most common first metastatic site was liver (*n* = 17), followed by lung (*n* = 16), non-regional lymph nodes (*n* = 11; including CI/PA), bone (*n* = 2), kidney (*n* = 1), and peritoneal (*n* = 1). Even though not all DR had been confirmed by biopsy, a retrospective assessment of the information in the medical records gave no indication of any false positive cases. At last follow-up, 21 of these 34 patients had died from anal cancer, 9 were alive with palliative treatment, and 4 were free of recurrence following curative intent treatment for oligometastatic disease.

### Common iliac and Para-aortic lymph node metastasis

CI/PA recurrences are included in the distant recurrences detailed in the section above, but are also described separately in the following. At a median of 13 months (range 5–81 months) from the date of diagnosis, 7 of 166 patients (4.2%; excluding patients with metastatic CI/PA nodes at initial diagnosis) had a CI/PA recurrence; 5 without other distant metastasis, and 2 with synchronous liver or lung metastasis. Significant associations were found between CI/PA recurrence and lymph node metastasis in ≥3 lymph node regions (*P* = 0.02), external iliac metastasis (*P* = 0.04), and T4 tumors (*P* = 0.045) (Table 2). Treatment of isolated CI/PA recurrence was salvage CRT in 4 patients, and at last follow-up, 2 of these were free of recurrence (at 25 and 26 months after CI/PA recurrence).

**Table 2:** Common iliac and/or para-aortic (CI/PA) recurrence in different subgroups

	CI/PA recurrence^a^		
	No, *n* (%)	Yes, *n* (%)	*P*-value^b^
**T stage**^c^			0.045
T1–3	126 (98)	3 (2)	
T4	33 (89)	4 (11)	
**N stage**^c^			0.44
0.	80 (98)	2 (2)	
1.	79 (94)	5 (6)	
**Lymph node regions**^d^**with metastasis**			0.02
< 3 regions	145 (97)	4 (3)	
≥ 3 regions	14 (82)	3 (18)	
**Site of lymph node metastasis**			
Inguinal	58 (94)	4 (6)	0.43^e^
Internal iliac	17 (90)	2 (10)	0.18^e^
External iliac	17 (85)	3 (15)	0.04^e^
Mesorectal^f^ or presacral	27 (93)	2 (7)	0.35^e^
**Distance from GTVN to iliac bifuration**^g^			0.32
< 5 cm	17 (90)	2 (10)	
≥ 5 cm	62 (95)	3 (5)	

^a^4 patients with CI/PA metastasis at diagnosis excluded

^b^Fisher’s exact test, 2-sided

^c^TNM8

^d^7 regions: left inguinal; right inguinal; left internal iliac; right internal iliac; left external iliac; right external iliac; mesorectal/presacral

^e^*P*-value for comparison with all other patients, including N0 patients

^f^Superior rectal included in mesorectal

^g^Distance from superior border of the most cranial lymph node metastasis to the bifurcation of common iliac artery

Based on the assumption that CI/PA metastasis at the time of initial diagnosis should be considered a potentially curable disease, curative intent CRT has been standard of care at our institution over the last years. Four such patients were included in the present study population. They all had pelvic nodal metastases as well, and all of them completed CRT (CTCAE v 5.0 acute gastrointestinal toxicity: no grade 3–5; two grade 2). With a median follow-up of 21 months (range 11–75 months), none of them had experienced a recurrence.

### Other regions of special interest

**Fig. 3:**
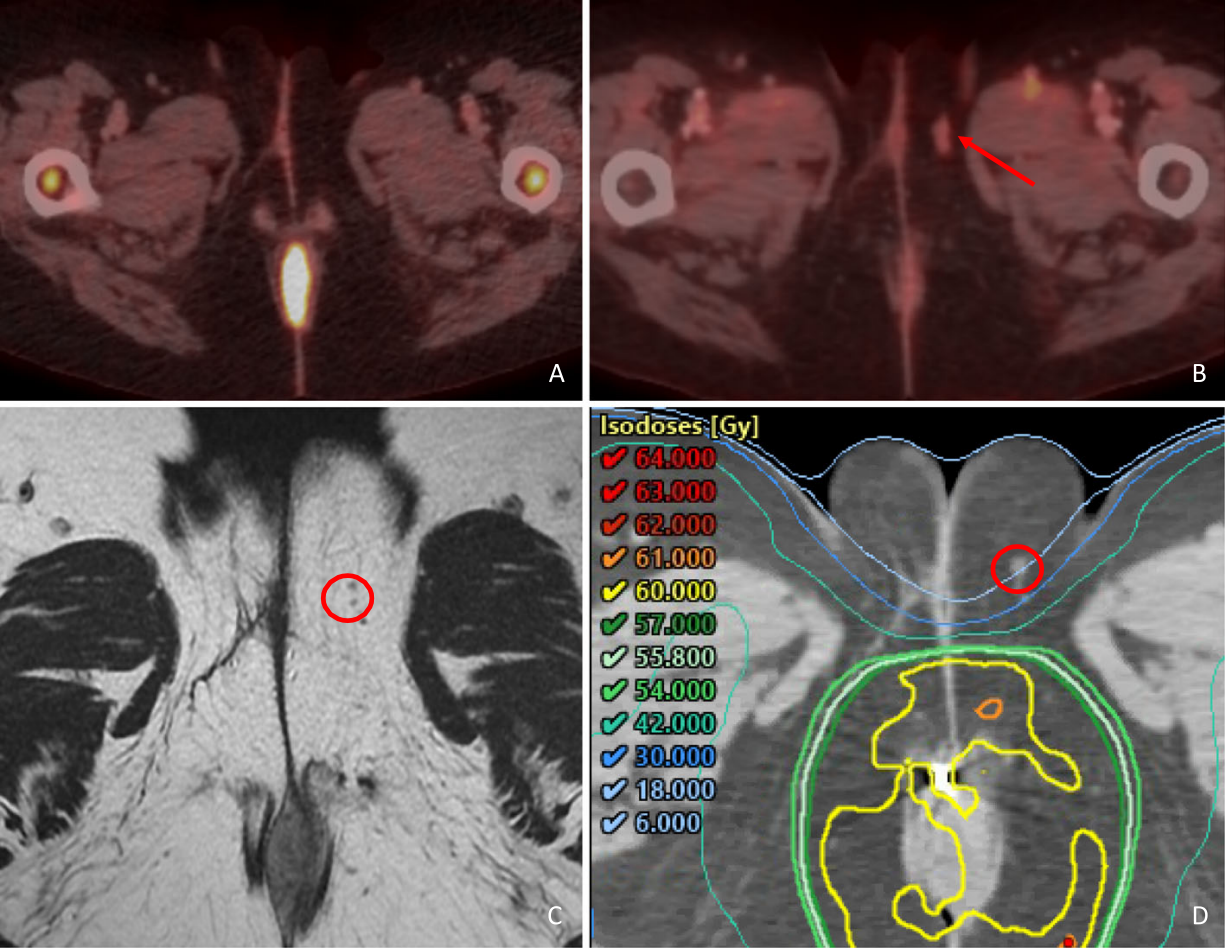
AILD recurrence

The AILD was not deliberately included in the elective CTV for the patients in the present study population. However, due to PTV expansions, and the nature of IMRT, some dose was delivered to parts of the AILD [13]. Two AILD recurrences were seen, both in patients with ipsilateral inguinal lymph node metastasis at the time of diagnosis (Fig. 2). Both patients also experienced an inguinal recurrence, indicating that the tumors were radioresistant. The overall rate of AILD recurrence was 2 of 170 (1.2%), and among patients with inguinal metastases at the time of diagnosis it was 2 of 65 (3.1%). Figure 3 shows one of the AILD recurrences.

Sacral hollows were not included in the elective CTV for 157 patients; none of whom experienced a recurrence located within a sacral hollow.

**Fig. 4:**
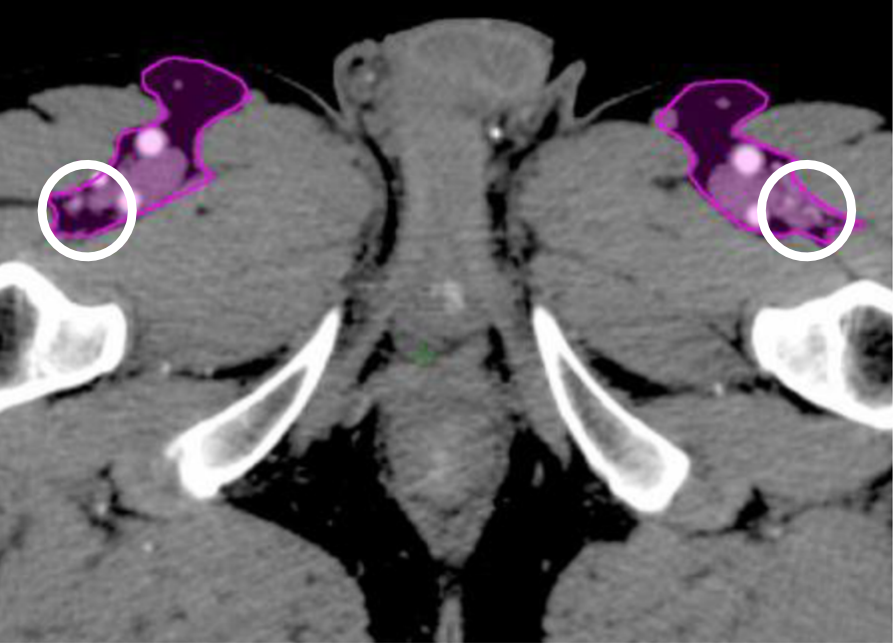
UK contouring guidelines with the inguinal area posterolateral to the deep vessels marked with a ring

International guidelines recommend elective coverage of the inguinal area posterolateral to the deep vessels (Fig. 4) [7–9]. In our cohort, 38 patients had < 50% of this area covered by the elective CTV; none of whom experienced a recurrence located in that area.

The center of the most inferior inguinal recurrence was located 1 cm below the saphenous junction (Fig. 2). Among 81 node negative patients who received elective coverage of the inguinal area, no inguinal recurrences were seen below the inferior border of the elective CTV, which was located 0–19 mm distal to the saphenous junction in 18 patients, 20–29 mm distal to the saphenous junction in 22 patients, and ≥ 30 mm distal to the saphenous junction in 41 patients.

## Discussion

This study investigated patterns of recurrence in a relatively large cohort of anal cancer patients treated with modern radiotherapy techniques. The most salient findings were: (1) out-of-field LR and isolated RR were very infrequent; (2) patients with metastatic lymph nodes in the external iliac area or in ≥3 lymph node regions had a relatively high risk of CI/PA recurrence; (3) some patients with CI/PA metastasis were free of recurrence following CRT; (4) DR was more common than LRR; (5) AILD recurrence occurred in 3.1% of the patients with metastatic inguinal lymph nodes at initial diagnosis.

Only one LR was a located outside the high dose (CTVT) volume. In other words, 169 of 170 patients did not have an out-of-field GTVT recurrence. The retrospective nature of our study precludes any firm conclusions, but the results suggest that a larger margin than 15 mm from GTVT to CTVT is not necessary, in line with current UK (10–15 mm), rather than Australian (20 mm) recommendations [7, 9]. Furthermore, other measures than increased margins are needed to improve local control.

Despite a small number of events, we were able to show that external iliac lymph node involvement (*P* = 0.04), and metastases in ≥3 inguinal or pelvic lymph node regions (*P* = 0.02) were associated with a 15–18% risk of CI/PA recurrence. A risk of recurrence exceeding 10–15% in a lymph node station is commonly considered high enough to include that station in the elective CTV [10]. If our exploratory results are replicated by others, the risk of CI/PA recurrence might be sufficiently high for these subgroups to merit inclusion of the CI/PA lymph node stations in the elective CTV.

Another important goal for anal cancer research should be to identify patients who could safely be treated with a smaller elective CTV than what is currently recommended. Hampered by the retrospective nature of the study, our results still lend some support for the opinion that the inguinal area posterolateral to deep vessels, as well as the sacral hollows, could be omitted from the elective CTV for most patients.

Holliday et al. reported outcomes for 30 anal cancer patients with metastatic PA lymph nodes at initial diagnosis. Half of the patients experienced no recurrence following curative intent up-front CRT. The authors concluded that extended-field CRT is a potentially curable treatment option for these patients [21]. We agree, and believe that the results of our present study corroborate their statement – 4 of 4 patients with CI/PA metastasis at initial diagnosis, and 2 of 5 with CI/PA recurrence, were free of recurrence at last follow-up. To avoid the risk of wrongly treating these patients with palliative instead of curative intent, and to emphasize the regional rather than distant pattern of spread, metastatic CI/PA lymph nodes might more properly be staged N2–3 than M1 disease.

The rate of LRR in our cohort compares favorably with other studies [1, 2, 22–31]. However, the rate of DR is higher than in most other studies, and the ratio between DR and LRR is higher in our study than in any other study. Using the same statistical method as comparable studies have used to estimate cumulative incidence (Kaplan-Meier), our 3-year LRR was 13.4% and our 3-year DR was 19.2%. For comparison, Shakir et al. recently reported a 3-year LRR of 19.2% and a 3-year DR of 10.9%, in a series of anal cancer patients treated according to UK guidelines [31]. While the pattern of LRR in our study is very similar to the pattern of LRR in the study by Shakir et al. – with a majority of LRR being in-field LR, and isolated RR being very infrequent - we have no clear explanation for the different ratios between DR and LRR. It could be a chance finding, but could also be attributed to methodological (length of follow-up, data collection method, definition of endpoints), biological (rates of HPV- and HIV-associated tumors), and treatment-related differences (radiation dose, systemic treatment) between the study cohorts. Over the past decades, there has been a steady decrease of LRR, probably explained mainly by a combination of more effective radiotherapy and an increasing proportion of radiosensitive HPV-associated tumors [32–34]. In rectal cancer, the focus of novel treatment interventions has shifted from decreasing LRR to decreasing DR. It is possible that future anal cancer studies will evolve in a similar direction.

The major limitation of our study is the retrospective design. Inherent bias, which is hard to assess and impossible to eliminate, makes the results exploratory. Before any of our conclusions could be clinically implemented, the results need to be validated in other studies, preferably with prospective study designs. However, one should note that most of the current recommendations regarding anal cancer IMRT target delineation are based on expert opinion. Another limitation is the sample size. A well characterized cohort of 170 patients is not small for a rare disease like anal cancer, but still not large enough to provide robust point estimates on various outcomes.

## Conclusion

In conclusion, LRR was relatively rare in this contemporary anal cancer cohort, but DR was more frequent than in previous studies. A changing ratio of LRR versus DR might have implications for endpoints and interventions in future clinical trials. Recurrent or primary metastatic CI/PA lymph nodes should be considered a potentially curable disease. Patients with certain patterns of metastatic pelvic lymph nodes might be at an increased risk of harboring tumor cells also in the CI/PA lymph nodes.

## Supplementary information

**Additional file 1.** Supplementary Table S1. DFS according to TN stage.

## Data Availability

The present data is summarized in this paper.

## Abbreviations

ACSS: Anal cancer specific survival
AILD: Ano-inguinal lymphatic drainage
CRT: Chemoradiotherapy
CTV: Clinical target volume
CI/PA: Common iliac and/or para-aortic
DFS: Disease free survival
DR: Distant recurrence
GTV: Gross tumor volume
IMRT: Intensity-modulated radiotherapy
LR: Local recurrence
LRR: Locoregional recurrence
OS: Overall survival
RR: Regional recurrence
PTV: Planning target volume
SIB: Simultaneous integrated boost
