# Supplementary material for: Patterns of recurrence in anal cancer: a detailed analysis
Source: Radiat Oncol. 2020 May 27;15:125. doi: 10.1186/s13014-020-01567-7 (PMC7251738; doi:10.1186/s13014-020-01567-7)
Supplement: Supplementary file 1 — Additional file 1. Supplementary Table S1. DFS according to TN stage. [file 13014_2020_1567_MOESM1_ESM.pdf]

Supplementary Table S1. Disease free survival (DFS) according to TN stage

	3-year DFS (%)	5-year DFS (%)
T2N0 (*n* = 49)	81	75
T3N0 (*n* = 11)	72	48
T4N0 (*n* = 11)	55	41
T2N+ (*n* = 34)	66	66
T3N+ (*n* = 24)	88	67
T4N+ (*n* = 27)	66	61
T3-4N+ (*n* = 51)	76	64
T3-4 (*n* = 73)	72	58
N0 (*n* = 82)	75	66
N+ (*n* = 88)	73	65
